# The Micro-Organism of Distemper in the Dog, and the Production of Distemper Vaccine

**Published:** 1901-12

**Authors:** S. Monckton Copeman


### SELECTIONS FROM OUR EXCHANGES.

### "THE MICRO-ORGANISM OF DISTEMPER IN THE DOG, AND THE PRODUCTION OF DISTEMPER VACCINE."

By S. Monckton Copeman, M.A., M.D., F.R.C.P.

[Communicated by Sir M. Foster, Sec., R. S. From the Proceedings of the Royal Society, vol lxvii.]

(*From the Brown Institution.*)

Distemper is so fatal a disease of dogs, more particularly of such as are highly bred, that a method of preventing invasion by the disease has always been a desideratum.

As the result of investigations into the bacteriology of this disease, carried out in continuance of those commenced in my laboratory at St. Thomas' Hospital about ten years ago by the late Everett Millais, I find that the specific microörganism concerned is a small coco-bacillus which stains with the ordinary aniline dyes, but is decolorized by the method of Gram. It grows readily on the surface of agar at body temperature; the individual colonies, when isolated by the method of plate-culture, having a grayish, glistening, semi-translucent appearance by reflected light, and a light brownish tint by transmitted light. The general form is circular; but occasionally, and especially in primary growths, the edge is somewhat irregular. The microbe also grows well in beef-broth, causing at first a general turbidity. Later on a deposit falls to the bottom of the tube, and the supernatant liquid becomes somewhat clearer. In cover-glass preparation from broth-cultures the bacilli are not infrequently found united together to form chains, sometimes of considerable length. The bacillus is capable of growing, though comparatively slowly, on solidified blood-serum and also in milk, which does not become coagulated. On potato it develops with difficulty, but now and again, after some days' incubation, a moist-looking streak of a pale buff color may be observed. If gelatin be inoculated, growth occurs slowly at the temperature of the room, and, after a time, the medium tends to become liquefied.

Growth on agar can be carried on week after week for a great number of generations, but after a dozen removes or so its morphological and biological characteristics are found to have become somewhat altered. An account of these variations and of the pathological histology of the disease I propose to publish subsequently.

In similar fashion the pathogenetic properties of the micro-organism appear to become gradually weakened, but by repeated intra-peritoneal inoculations in the guinea-pig its virulence may be regained.

The injection of 1 c.c. of a broth-culture seven days old, derived in turn from an agar subculture, beneath the skin of the abdomen in a dog weighing seven kilos, induced an attack of distemper, which terminated fatally in about a week from the time of inoculation. In a large number of other dogs experimented on by Millais or myself a generally non-fatal attack has followed on inoculation of the nasal mucous membrane.

Specially characteristic of the disease intentionally produced is the fact that the animal exhibits during the attack a marked and progressive loss of weight. Of other symptoms of the malady so well known to all dog-breeders, those which are usually most marked are the result of more or less acute inflammation of the various mucous surfaces.

On post-mortem examination I have generally found the whole respiratory tract to be specially affected, the lungs sometimes showing pneumonic consolidation throughout almost their entire extent. The trachea is apt to be congested and to contain a quantity of mucus, while the eyes and nose are blocked with a purulent or mucopurulent discharge. By making agar plate cultivations from the exudation from the lungs, from the tracheal mucus, or from the nasal secretion, the specific bacillus may be isolated from the first two situations, often in almost pure culture.

Examining animals which have died from distemper, whether resulting from experimental inoculation or contracted in the ordinary fashion, I have never succeeded in obtaining cultures either from the blood obtained from the heart with aseptic precautions, or from the liver, the gall-bladder, the kidney, or the spleen. Pressure of other work since joining the Medical Staff of the Local Government Board has prevented my having the opportunity of examining even inoculated animals at intermediate stages of the disease in severe forms, or, doubtless, it might have been found possible to isolate the bacillus from one or other of these situations. In one instance, in which the bloodvessels of the brain were found to be much congested, inoculation of a tube of sloping agar with a large platinum loopful of cerebro-spinal fluid, well spread over the surface of the agar, resulted in the appearance of half a dozen isolated colonies of a pure culture of the distemper bacillus.

By heating a broth-culture of the bacillus at 60° C. for half an hour, and subsequently adding a small quantity of carbolic acid as a preservative, a vaccine is obtained which acts in a similar fashion to those devised by Haffkine and Wright for use in the prevention of plague and enteric fever, respectively. The vaccine may be standardized after the manner originally suggested by Wright in connection with his work on enteric fever.

The dose must obviously vary according to the size of the dog, but, as a guide, it may be mentioned that I have found, in three instances, that the injection of 2 c.c. of the sterilized culture of the bacillus is apparently sufficient to protect fox-terrier puppies weighing about one and a half kilos against attack by distemper, while an unprotected puppy in the same batch contracted the disease on introduction of an affected dog. I also find that guinea-pigs can be protected in this way against the effects of a dose of living culture which would ordinarily prove fatal in about forty-eight hours. As regards the exact length of time, however, during which such protective effect may last, no definite statement can as yet be made, but a series of tests on a large scale are in process of being carried out by dog-breeders in this country, in Germany, and in America.—*The Veterinary Record*, November 16, 1901.

